# Intravenous streptozotocin induces variants in painful diabetic peripheral neuropathy in female mice

**DOI:** 10.21203/rs.3.rs-6058617/v1

**Published:** 2025-02-21

**Authors:** Michelle Hagenimana, Audrey R. Green, Jessica A. Tierney, Melissa E. Lenert, Michael D. Burton

**Affiliations:** 1 Laboratory of Neuroimmunology and Behavior, Department of Neuroscience, Center for Advanced Pain Studies (CAPS), School of Behavioral and Brain Sciences, University of Texas at Dallas 800 W. Campbell Road, Richardson, TX 75080, United States

**Keywords:** Sex differences, females, STZ, males, diabetic peripheral neuropathy, hyperglycemia, Glucose tolerance test

## Abstract

**Purpose:**

At least half of the 34 million diabetic patients in the U.S. develop painful diabetic peripheral neuropathy (DPN). Recent evidence suggests that there are sex differences in the prevalence and mechanisms underlying pathological pain states. However, due to technical limitations in murine models, there is a dearth of the use of females in diabetic neuropathy research. There is a need to develop a reproducible model to induce painful DPN in both sexes. The predominately used model employs streptozotocin (STZ) administration via intraperitoneal injections. This strategy consistently induces diabetic pathology in males, but not females. We set out to enhance a current murine DPN model by identifying a method for inducing diabetic pathologies in male and female mice and tracking the development of painful neuropathy.

**Methods:**

Painful DPN was induced in both male and female mice through five daily intravenous injections of STZ. Metrics of diabetic pathology and pain behaviors were assessed across the development of painful DPN. To investigate neuronal sensitivity, calcium imaging assessing dorsal root ganglia neurons was performed.

**Results:**

We found that after intravenous administration of STZ, both male and female mice exhibited elevated blood glucose levels, impaired glucose tolerance, and increased mechanical and thermal hypersensitivity. Interestingly, we observed a subset of STZ-insensitive animals that displayed comparable elevated glucose levels to the STZ-sensitive animals. Calcium imaging was performed to investigate neuronal sensitivity and we found that both male and female mice with diabetic pathology had a lower latency to peak capsaicin-induced response compared to their control counterparts.

**Conclusion:**

Our findings demonstrate that intravenous administration of STZ can induce comparable diabetic neuropathy in male and female mice and opens the door for future preclinical studies on diabetic neuropathy.

## Introduction

Despite evidence of females having an increased prevalence of chronic pain in clinical populations^1^, preclinical studies have been slow to include females in pain research^2,3^. Recent initiatives have urged and incentivized the consideration of sex as a biological variable in preclinical research and results from recent preclinical studies in chronic pain further assert the relevance and importance of including sex as a biological variable^4,5^.

Diabetic peripheral neuropathy (DPN) is common complication of diabetes. It is a chronic heterogeneous disease that most commonly presents in the extremities (hands and feet) as a distal sensorimotor neuropathy. Symptoms can include spontaneous (stimulus-independent) burning or stabbing pain and feeling of electrical shocks, which are associated with small fiber neuropathy, or numbness, tingling, and gait instability, which are associated with large fiber neuropathy^6^. As such, DPN can be further characterized as painful or nonpainful DPN^7,8^. Painful DPN is characterized by burning and/or tingling sensations and develops in approximately 30% of diabetic patients ^9–12^. There is also a prevalence of nonpainful DPN, which is characterized by a loss of pain, heat, cold or pressure sensation^6,13,14^. Although the exact cause of DPN is yet to be determined, there are several risk factors associated with the development of DPN, including obesity, elevated fasting glucose or glycosylated hemoglobin A1c (HbA1c) indicative of poor glycemic control, and duration of diabetes^15^. Studies have also suggested that the female sex may also be a risk factor for the development of DPN, especially painful DPN^12^.

To study the development of DPN, researchers have employed preclinical animal models. There is no standardized definition of diabetes in mice; therefore, to characterize the presence of diabetic pathology, measurements such as body weight, blood glucose concentration, and glucose tolerance are observed longitudinally and compared to a baseline and a control group. Blood glucose concentrations are used to observe glucose homeostasis and are analogous to hyperglycemic measurements used in clinical settings^16^. The glucose tolerance test is used to observe impaired glucose metabolism and homeostasis and is analogous to the glucose tolerance test used in clinical diagnostic settings^17^. Hyperglycemia is widely accepted to play a causative role in the development of DPN. Thus, glycemic control is used as a means of therapeutic intervention for diabetic comorbidities in humans and assessment of hyperglycemia can be used as a measure of DPN development in mice.

One popular chemically induced model of diabetes and DPN uses streptozotocin (STZ). STZ is a nitrosourea analogue that enters pancreatic beta cells via the GLUT2 transporter to ablate beta cells and cause the subsequent loss of insulin production by the pancreas in a manner mechanistically similar to type-1 diabetes mellitus (T1DM)^18^. Although this model is efficient at inducing diabetes in male mice, STZ administration has been largely ineffective at inducing diabetic pathology in female mice^16,19–22^. Although the female sex has been implicated as a risk factor for the development of DPN, preclinical research on DPN has historically been male-biased due to STZ’s ineffectiveness in females^23^. This partial resistance can be attributed to the protective effect of estrogen and the metabolism of STZ due to its short half-life^20,24,25^. In order to assess DPN in females, an efficient and effective model to induce DPN in females is needed.

We set out to establish an effective method of inducing DPN in male and female mice using intravenous (i.v.) STZ^26–29^. STZ is typically administered by intraperitoneal injections, but i.v. injections have previously been found to induce an equivalent diabetic phenotype^26^. However, the diabetic phenotype induced by i.v. STZ injection was characterized in male mice because female mice were previously found to be less sensitive to STZ^26^. Therefore, we measured blood glucose levels in male and female mice following STZ injection and performed evoked behavioral measures of pain to mechanical and thermal stimuli to characterize the development of DPN. Some rodent models have recapitulated painful and nonpainful DPN pathologies separately, with painful DPN developing shortly after the induction of diabetic pathology and nonpainful DPN developing later^30,31^. Here, we were able to associate “high” vs. “low” responders to the STZ to differentiate painful vs. nonpainful subjects. We were also able to concomitantly differentiate both painful and nonpainful DPN animals based on their fed glucose levels and correlate that to the severity of mechanical hypersensitivity, similar to clinical populations.

Beyond behavioral pain phenotypes, physiological biomarkers are also characteristic of DPN. Transient Receptor Potential Vanilloid type (TRPV1) ion channels are heat sensitive cation channels expressed on dorsal root ganglion (DRG) sensory neurons that respond to noxious heat^32^. TRPV1 may be modulated in STZ models of painful DPN such that upregulation of TRPV1 may contribute to thermal hyperalgesia in painful DPN while downregulation of TRPV1 may contribute to hypoalgesia in nonpainful DPN^30,33^. We used calcium imaging to elucidate whether STZ induced DPN altered TRPV1 mediated Ca^2+^ responses in DRG sensory neurons in both sexes which may contribute to the development of painful DPN.

## Materials and methods

### Animals

All animal procedures were done in accordance with protocols approved by the University of Texas at Dallas Institutional Animal Care and Use Committee (Protocol 17–10). All experiments were performed using male and female mice (age ranged 2–6 months). We used wild-type C57 animals (Stock No. 000664) purchased from Jackson Lab and then bred in-house and used as the background strain to all transgenic animals. Transgenic Pirt-GCaMP3 and Pirt-cre animals were a gift from Xinzhong Dong (Johns Hopkins)^34^, and lox-stop-lox (LSL) Salsa6 transgenic reporter animals were purchased from Jackson Lab (Stock No. 031968). Pirt-GCaMP3 allows for the expression of GCaMP3, a genetically encoded calcium indicator which fluoresces when calcium is bound to be expressed on DRG neurons under the Pirt promoter and allows for assessment of intracellular calcium concentrations^32^. Salsa6 transgenic reporter animals have a tdtomato linked to GCaMP6f and tdtomato was used to identify cells that express GCaMP6f for calcium imaging experiments. Salsa6f were crossed with Pirt-cre mice and used for behavior and calcium imaging experiments. Once received, mice were bred in-house. All strains were backcrossed at least 8 generations to a C57BL/6 background. The mice were weaned, and tail clipped between 21 and 28 days of age to verify genotype. Animals were group housed in polypropylene ventilated cages with 3–5 mice per cage with ad-libitum access to water and regular chow diet. Room temperature was maintained at 21 ±2°C and the facility maintained a 12-hour light/dark cycle (lights on 0700h/lights off 1900h).

### Induction of Diabetic Pathology

Diabetic pathology was induced by i.v. injection of streptozotocin (Sigma-Aldrich, S0130–500MG) dissolved (8 mg/ml) in a sodium citrate (Sigma-Aldrich, C8532–500G) buffer (18.35 mg/mL, pH 4.5) for five consecutive days, at 50 mg/kg each day. Mice were fasted for 4 hours prior to i.v injections and split into two treatment groups; one group was administered STZ freshly dissolved in sodium citrate buffer, and the other was administered sodium citrate buffer as a control. For the duration of the 5 days, all cages were provided with 10% sucrose-water solution following the injections to prevent fatal hypoglycemia^26,35^. Fed blood glucose levels were measured, as detailed below, to assess the induction of diabetes (fasting glucose levels ≥200 mg/dL).

### Fed Blood Glucose Measurements

All glucose testing was performed in a quiet environment, during the light cycle (between 1300h and 1700h). Mice were fasted in a clean cage, with an empty food hopper, for 4 hours prior to glucose testing (between 0900h and 1300h). While animals can be fasted overnight (14–16 hours) prior to testing to obtain a fasted blood glucose measurement, this is not indicative of glucose homeostasis in a free-living state but rather in a state of metabolic stress^36^. Alternatively, animals can be fasted 4–6 hours prior to glucose testing to obtain a fed glucose measurement that is indicative of basal glucose levels. After fasting, blood was quickly taken from the tip of the tail, and blood glucose levels were measured using an AlphaTrak 2 Veterinary Blood Glucose Monitoring Meter Kit. Glucose testing was performed 12, and 16 days after the last STZ injection to assess STZ-induced diabetes.

### Glucose Tolerance Test

For the glucose tolerance test, the mice were fasted for 16 hours, after which baseline blood glucose measurements were taken using an AlphaTrak 2 Veterinary Blood Glucose Monitoring Meter Kit. Following the baseline measurements, the mice were injected intraperitoneally with a 2g/kg dose of glucose (Sigma, G7528–250G). Blood glucose levels were measured at 20, 40 60, 80, 100, and 120 minutes after glucose administration.

### Behavioral Testing

Before baseline measurements, animals were acclimated to the testing room, which was at a temperature of 21 ±2°C. On behavioral testing days, animals were habituated in acrylic behavior boxes on an elevated wire mesh grid for one hour prior to testing. Two baseline behavioral measurements were taken prior to the STZ injections and afterwards animals were randomly assigned to treatment groups. Behavioral assessments for mechanical hypersensitivity and thermal hypersensitivity were performed 6, 9, 12, 16, 19, and 21 days after the last STZ injection. All behavior assessments were performed by an experimenter blinded to treatment condition.

### Mechanical Hypersensitivity

To measure mechanical hypersensitivity, animals were placed in elevated acrylic behavior racks, comprised of individual transparent acrylic compartments 11.43 cm in length and 5.08 cm in width with a wire mesh floor. Animals were habituated on the behavior rack for approximately 2 hours before behavioral testing began. Mechanical hypersensitivity was measured as paw withdrawal threshold (PWT) using von Frey filaments (Stoelting, 58011) and the up-down experimental method.^37^ Von Frey filaments were applied to the plantar surface of both hind paws using the up-down method, and withdrawal thresholds from both hind paws were averaged together to obtain the plotted PWT values.

### Thermal Hypersensitivity

Thermal hypersensitivity was measured as paw withdrawal latency and was assessed using the hot plate apparatus (IITC, California, USA). Animals were placed on a surface maintained at 4°C to assess cold sensitivity. Animals were maintained in the apparatus until a positive response was observed (e.g., jumping, hind paw licking, and shaking). Paw withdrawal latency was measured as the time spent in the apparatus until the positive response was observed. The time for assessment was capped at 30 seconds to prevent injury to the animals. Mechanical hypersensitivity measures were always taken before thermal hypersensitivity was measured.

### Primary Dorsal Root Ganglion (DRG) Culture

Mice were deeply anesthetized with isoflurane and euthanized by decapitation. DRGs from all levels were dissected and were transferred to chilled HBSS (Hanks’ Balanced Salt Solution without calcium or magnesium) (Gibco, Cat#14–170-112). The DRG’s were digested using collagenase A (1:1; A (Sigma-Aldrich, Cat#10103586001): HBSS) for 25 minutes at 37°C, and collagenase D (1:1:10%; D (Sigma-Aldrich, Cat#1188866001): HBSS:papain (Sigma-Aldrich, Cat#10108014001)) for 25 minutes at 37°C. The tissue was put in a trypsin inhibitor solution (1:1:1; Trypsin (Sigma-Aldrich, Cat#10109886001): BSA: TG Media) to stop the enzymatic digestion. The digested tissue was mechanically homogenized via trituration, and then passed through a 70-μm nylon cell strainer (Millipore Sigma, Z742103–50EA). The cells were pelleted and resuspended in TG media (DME/F12 1:1 with 2.50 mM L-Glutamine and 15 mM HEPES buffer (HyClone) supplemented with 10% Fetal bovine serum (HyClone, Cat#SH30088.03) and 1% Penicillin Streptomycin (Fisher Scientific, Cat#15070063)). The cells were plated on 35 mm petri dishes, 10 mm Microwell, and No. 1.5 cover glass (MatTek Corporation, P35G-1.5–10-C) that were coated with a 200 μg/mL poly-D lysine solution (Sigma-Aldrich, Cat#P0899); 50 μL of the cell suspension were plated on the center of the plate. The plates were incubated at 37°C with 5% CO_2_ for 2 hours, and then flooded with 2–3 mL of TG Media. The plate was incubated at 37°C with 5% CO_2_ and calcium imaging was done the next day.

### Calcium Imaging

Calcium imaging experiments were performed using tissue samples from Pirt-GCaMP3 transgenic mice and Salsa6 transgenic reporter mice. 30 minutes before imaging, TG Media was removed and replaced with bath solution (125 mM NaCl (Fisher Scientific, Cat#S271–500), 5 mM KCl (Fisher Scientific, Cat#P217–500), 10 mM HEPES (Sigma-Aldrich, Cat#H4034), 1000 mM CaCl2 (Sigma-Aldrich, Cat#21115, 1,000 mM MgCl2 (Fisher Scientific, Cat#M35–500), and 2,000 mM glucose (Sigma-Aldrich, Cat#G7528)) at a pH of 7.4 ± 0.05 and mOsm of 300 ± 5 at 37 °C. Calcium Imaging was performed using the MetaFluor Fluorescence Ratio Imaging Software on an Olympus TH4–100 apparatus and imaging was performed with a 40× objective on the FITC channel. Changes in GCamp3 and GCamp6 fluorescence intensity were recorded in real time. Baseline measurements were taken for 30 seconds, during which bath solution was applied. Capsaicin (Sigma-Aldrich, Cat#M2028) (250 nM), a known TRPV1 agonist that has been shown to mediate DPN hypersensitivity, was used during calcium imaging to assess the impact of DPN on neuronal activity ^33^. Following baseline measurements, capsaicin was applied for 20 seconds. After capsaicin application, the bath solution was applied for a 120 second wash and was followed by a 10 second application of 50 mM KCl, which was used as the positive control to assess neuronal response. Any cell with an increased response from baseline of at least 20% during KCl perfusion was considered a neuron, and only neurons were included in data analysis. Any cell with an increased response from baseline of at least 16% during capsaicin perfusion was considered responsive to capsaicin.

### Statistical Analysis

Graphpad Prism 9 (GraphPad, San Diego, CA, USA) was used to generate all graphs and statistical analysis. Fed glucose levels, weight assessments and the glucose tolerance test were analyzed using three-way ANOVA with Tukey’s *post-hoc* test. Behavioral data at each time point was analyzed using two-way mixed analysis with Tukey’s *post-hoc* test. Calcium imaging data and effect size were analyzed using two-way ANOVA with Sidak’s multiple comparisons test. The percentage of capsaicin responsive cells from the calcium imaging experiment was analyzed using Fisher’s exact test. For all statistical analysis, *p*<0.05 was considered statistically significant. All data are represented as mean and the standard error of the mean (mean ± SEM).

## Results

### I.v. administration of STZ induces hyperglycemia and impaired glucose tolerance in males and females

**Figure 1:**
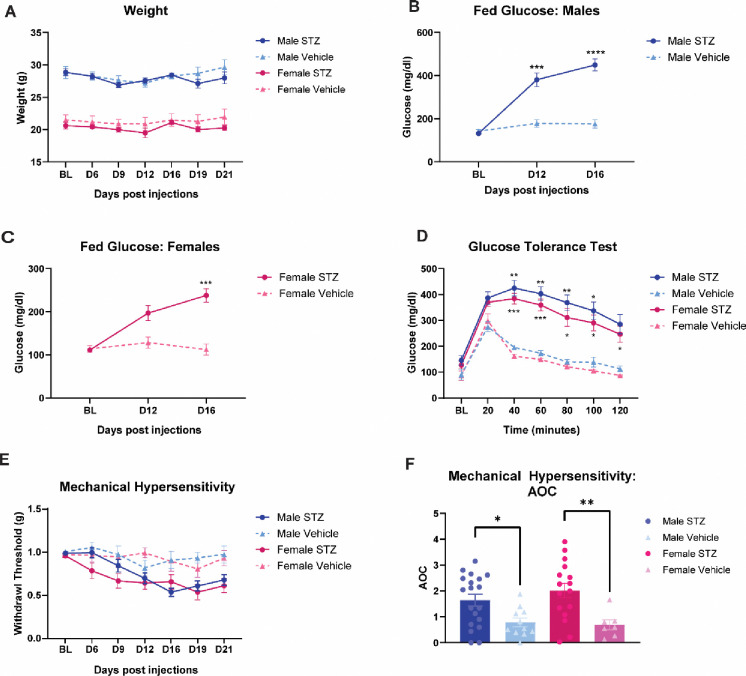
I.V. administration of STZ induces hyperglycemia and impaired glucose tolerance in males and females. (**A**) the assessment of STZ on mouse body weight (**B**) the effects of STZ on fed glucose levels in male mice (**C**) the effects of STZ on fed glucose levels in female mice (**D**) utilization of the glucose tolerance test to assess for STZ-induced impaired glucose tolerance in males and females (**E**) male and female mechanical hypersensitivity following STZ administration (**F**) area over the curve analysis of mechanical hypersensitivity in male and female STZ- or vehicle-treated mice. *p<0.05, **p<0.01, ***p<0.001 vehicle vs STZ.

To assess STZ-induced diabetic pathology, total body weight, fed blood glucose levels, and glucose tolerance were measured (Figure 1). STZ administration had no significant effect on the weight development of males or females (Figure 1A). To assess STZ-induced hyperglycemic pathology, fed blood glucose levels were measured at 12- and 16-days post STZ injections. In both males and females, STZ administration significantly increased fed glucose levels (F (1, 51) = 43.52, *p*< 0.0001) (Figure 1B and C). Additionally, male mice had an earlier onset of hyperglycemic pathologies in comparison to females, with males showing a significant difference between STZ and vehicle treated groups at 12 days post STZ administration (*p*=0.0003) and females showing a significant difference by day 16 *(p*=0.0002) (Figure 1B and C).

To further assess STZ-induced diabetic pathology, a glucose tolerance test was performed 22 days post-STZ injections (Figure 1D). Following a 16 hour fast, mice were administered a 1g/kg dose of glucose and their blood glucose levels were measured across 2 hours. Regardless of sex, STZ had a significant effect on blood glucose levels (F (1, 51) = 43.52, *p*< 0.0001), with STZ-treated animals having higher glucose concentrations compared to their vehicle counterparts. Taken together these findings indicate that STZ-administration induced hyperglycemia and resulted in impaired glucose metabolism in male and female mice.

To assess STZ-induced diabetic neuropathy, mechanical hypersensitivity was measured using von Frey filaments up to 21 days post STZ injections (Figure 1F). Animals administered STZ had significantly lower paw withdrawal thresholds (F (1,50) = 26.41, *p*<0.0001), indicating the presence of diabetic neuropathy (Figure 1F). Additionally, area over the curve (AOC) analysis showed that STZ-treated males and females were significantly more sensitive in comparison to their vehicle-treated counterparts (F (1, 49) = 15.98, *p*=0.0002), and there were no significant differences in mechanical hypersensitivity between males and females administered STZ (Figure 1F).

### STZ-induced diabetic pathology results in distinct mechanical hypersensitivity variants in males and females

**Figure 2:**
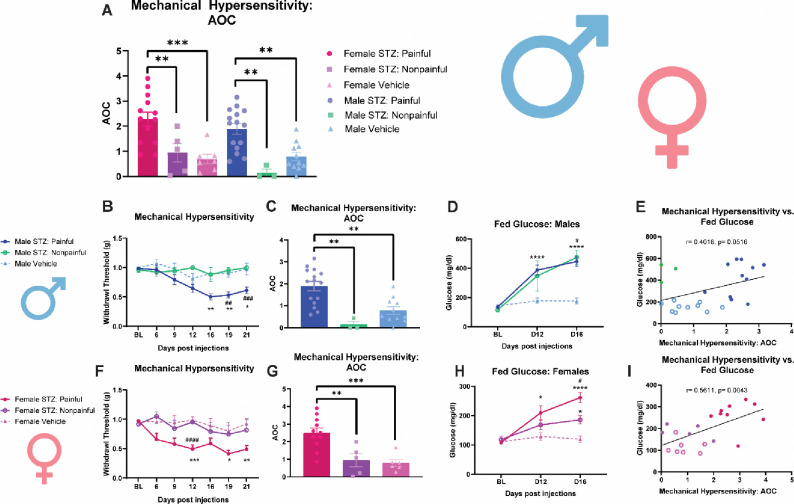
STZ-induced diabetic pathology results in distinct mechanical hypersensitivity variants in males and females. (**A**) area over the curve analysis of mechanical hypersensitivity in male and female STZ-induced painful DPN, STZ-induced nonpainful DPN, and vehicle mice **(B**) comparison of mechanical hypersensitivity of painful DPN, nonpainful DPN, and vehicle male mice following STZ injections (**C**) area over the curve analysis of mechanical hypersensitivity of painful DPN, nonpainful DPN, and vehicle male mice (**D**) fed glucose levels from painful DPN, nonpainful DPN, and vehicle male mice following STZ injections (**E**) Correlation of mechanical hypersensitivity (AOC) vs fed glucose levels (mg/dl) in painful DPN, nonpainful DPN, and vehicle male mice **(F**) comparison of mechanical hypersensitivity of painful DPN, nonpainful DPN, and vehicle female mice following STZ injections (**G**) area over the curve analysis of mechanical hypersensitivity of painful DPN, nonpainful DPN, and vehicle female mice (**H**) fed glucose levels from painful DPN, nonpainful DPN, and vehicle female mice following STZ injections (**I**) Correlation of mechanical hypersensitivity (AOC) vs fed glucose levels (mg/dl) in painful DPN, nonpainful DPN, and vehicle female mice. *p<0.05, **p<0.01, ***p<0.001, ****p<0.001 vehicle vs STZ-induced painful DPN. ^#^*p*<0.05, ^##^*p*<0.01, ^###^*p*<0.001, ^####^*p*<0.001 STZ-induced nonpainful DPN vs STZ-induced painful DPN.

In efforts to further understand the STZ-induced diabetic pathology, we parsed out the behavioral responses of the STZ male and female groups (Figure 2A). Specifically, males administered STZ displayed two distinct DPN variants: a more sensitive ‘painful DPN’ variant, and a non-sensitive ‘nonpainful DPN’ variant (Figure 2A). On day 19, the STZ-treated males with a painful DPN phenotype had a significantly lower withdrawal threshold compared to both the vehicle-treated males (*p*=0.0011), and the STZ-treated males with a nonpainful DPN phenotype (*p*=0.0013) (Figure 2B). These findings were conserved on day 21, with STZ-males with a painful DPN-variant having a lower withdrawal threshold than their vehicle treated counterparts (*p*=0.0189) and STZ-treated males with nonpainful-DPN (*p*=0.0002) (Figure 2B). AOC analysis showed that STZ-treated males with painful-DPN were more sensitive than both STZ-treated males with a nonpainful-DPN (*p*=0.0025) and vehicle-treated males (*p*=0.0022) (Figure 2C). Analyzing fed glucose levels within the context of these two DPN variants reveals that the presence of sensitivity does impact blood glucose levels (F (2, 29) =18. 97, *p*<0.0001) (Figure 2D). Males with painful DPN had significantly higher blood glucose levels on both day 12 (*p*<0.0001) and day 16 (*p*<0.0001) in comparison to the vehicle group, while males with nonpainful DPN only had significantly higher glucose levels on day 16 (*p*=0.0298) in comparison to the vehicle group (Figure 2D). Furthermore, there was no significant difference between fed glucose levels of males with a painful DPN pathology and a nonpainful DPN pathology on day 12 or 16 (Figure 2D). Correlation analysis in males showed a positive but nonsignificant relationship between paw withdrawal threshold effect size values and fed glucose levels (Figure 2E, R^22^= 0.4017 *p*=0.0516).

Females administered STZ also displayed a painful variant and a nonpainful variant (Figure 2A). Females displaying painful DPN had a significantly lower withdrawal threshold on day 19 (*p*=0.0191) and day 21 (*p*=0.0050) compared to vehicle-treated animals, while the females displaying nonpainful DPN showed no significant difference compared to vehicle treated animals on day 19 (*p*=0.9624) and day 21 (*p*=0.8387) (Figure 2F). On day 12, both painful (*p*=0.0001) and nonpainful (*p*<0.0001) females had significantly lower withdrawal thresholds than vehicle-treated females (Figure 2F). To further assess differences in the mechanical hypersensitivity between these groups, paw withdrawal threshold was graphed as AOC (Figure 2G). The STZ-treated females with a painful DPN pathology had a significantly lower paw withdrawal threshold compared to vehicle-treated animals (*p*=0.0002) and the STZ-treated females with a nonpainful DPN pathology (*p*=0.0056) (Figure 2G). Female fed glucose levels were graphed within the context of the two DPN variants, and this revealed the presence of two distinct hyperglycemia profiles (Figure 2H). Females with a painful DPN pathology had significantly higher glucose levels at day 12 (*p*=0.0239) and 16 (*p*<0.0001) compared to vehicle-treated counterparts (Figure 2H). Furthermore, on day 16, females with a nonpainful DPN pathology had significantly higher blood glucose levels compared to the vehicle treated mice (*p*=0.0129), and significantly lower glucose levels compared to the painful DPN group (*p*=0.013) (Figure 2H). Correlation analysis revealed a significant positive correlation between paw withdrawal threshold effect size values and fed glucose levels in female mice (Figure 2I, R^22^=0.5611, *p*=0.0043). This indicates that STZ induces two distinct hyperglycemic profiles in females and these profiles result in painful and nonpainful DPN pathologies.

### STZ-induced diabetic pathology results in thermal hypersensitivity

**Figure 3:**
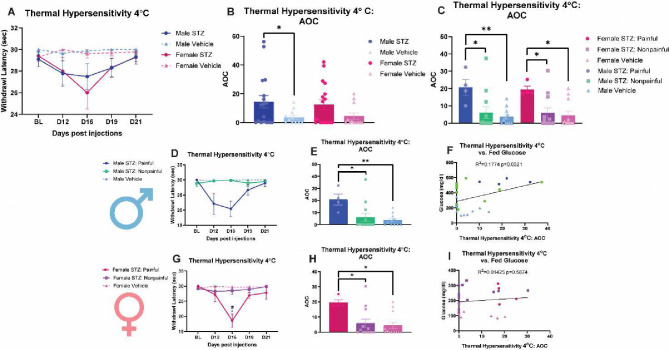
STZ-induced diabetic pathology results in thermal hypersensitivity. (**A**) male and female thermal hypersensitivity following STZ administration (**B**) area over the curve analysis of thermal hypersensitivity in male and female STZ- or vehicle-treated mice (**C**) area over the curve analysis of thermal hypersensitivity in male and female STZ-induced painful DPN, STZ-induced nonpainful DPN, and vehicle mice (**D**) comparison of thermal hypersensitivity of painful DPN, nonpainful DPN, and vehicle male mice following STZ injections (**E**) area over the curve analysis of thermal hypersensitivity of painful DPN, nonpainful DPN, and vehicle male mice (**F**) Correlation of thermal hypersensitivity (AOC) vs fed glucose levels (mg/dl) in painful DPN, nonpainful DPN, and vehicle male mice (**G**) comparison of thermal hypersensitivity of painful DPN, nonpainful DPN, and vehicle female mice following STZ injections (**H**) area over the curve analysis of thermal hypersensitivity of painful DPN, nonpainful DPN, and vehicle female mice (**I**) Correlation of thermal hypersensitivity (AOC) vs fed glucose levels (mg/dl) in painful DPN, nonpainful DPN, and vehicle female mice. *p<0.05, **p<0.01 vehicle vs STZ-induced painful DPN. ^#^*p*<0.05 STZ-induced nonpainful DPN vs STZ-induced painful DPN.

Thermal sensitivity was measured at 4°C to further understand the STZ-induced diabetic pathologies (Figure 3). There was no significant STZ-dependent effect on withdrawal latency in both sexes when all STZ responses were assessed together (Figure 3A). AOC analysis showed a significant effect of STZ on paw withdrawal latency (F (1, 53) =6.601, *p*=0.0130), but only in males (*p*=0.0430) (Figure 3B). When parsing out the STZ-induced painful and nonpainful variants, the AOC analysis showed that STZ-treated males and females with painful-DPN were more sensitive than both STZ-treated animals with a nonpainful-DPN and vehicle-treated animals (F (2, 49) =10.38, *p*=0.0002) (Figure 3C). No sex differences were observed when considering painful and nonpainful variants (Figure 3C).

Specifically, when separating the painful and nonpainful STZ variants in males, there were no significant changes in withdrawal latency in repones to the thermal stimuli (Figure 3D). The AOC analysis did indicate that STZ-treated males with painful-DPN were more sensitive than both STZ-treated males with nonpainful-DPN (*p*=0.0147) and vehicle-treated males (*p*=0.0045) (Figure 3E). Correlation analysis in males did show a significancy weak positive between thermal hypersensitivity and fed glucose levels (Figure 3F, R^2^=0.1774, *p*=0.0321).

In analyzing the painful and nonpainful STZ variants in females, a significant reduction in withdrawal latency was observed in the painful DPN group in comparison to both the nonpainful DPN (*p*=0.0243) and vehicle-treated (*p*=0.0277) animals on day 16 (Figure 3G). The AOC analysis did indicate that STZ-treated females with painful-DPN were more sensitive than both STZ-treated females with nonpainful-DPN (*p*=0.0295) and vehicle-treated females (*p*=0.0130) (Figure 3H). Correlation analysis in females did not show a relationship between thermal hypersensitivity and fed glucose levels (Figure 3I, R^2^=0.01425, *p*=0.5874).

### STZ-induced diabetic pathology results in faster neuronal response to capsaicin

**Figure 4:**
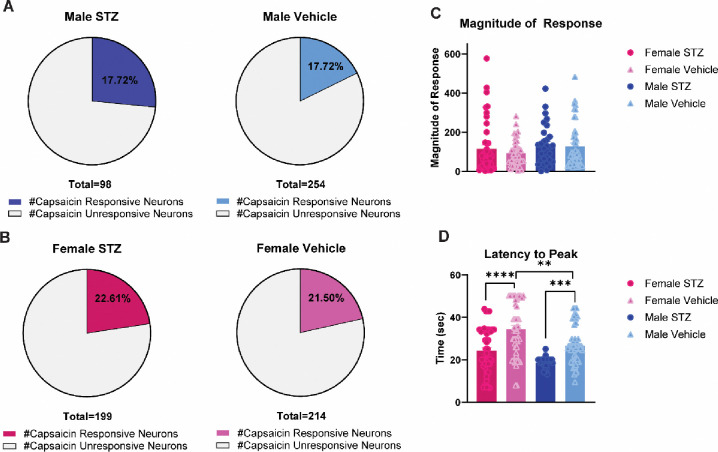
STZ-induced diabetic pathology results in faster neuronal response to capsaicin. (**A**) percent of capsaicin responsive DRG neurons from male STZ- or vehicle-treated male mice (**B**) percent of capsaicin responsive DRG neurons in male STZ- or vehicle-treated female mice (**C**) magnitude of response to capsaicin in DRG neurons from male and female STZ- or vehicle-treated mice (**D**) latency to peak in response to capsaicin in DRG neurons from male and female STZ- or vehicle-treated mice. **p<0.01, ***p<0.001, ****p<0.001 vehicle vs STZ.

Three weeks post-STZ administration, calcium imaging experiments were performed on cultured DRG neurons (Figure 4). STZ as a factor did not have a significant effect on the percentage of capsaicin responsive cells (Figure 4A and B) or the magnitude of response to capsaicin (Figure 4C) in males and females. STZ as a factor had significant effects on the latency to peak capsaicin response (**Figure 5D**, F (1, 164) = 39.42 *p*<0.0001). STZ-treated males and females had a significantly lower latency to peak response compared to their vehicle-treated counterparts (males; *p*=0.006, females; *p*<0.0001) (Figure 4D). There was a significant difference between male and female vehicle-treated animals, with males have a significantly lower latency to peak than females (*p*=0.0095) (Figure 4D). This indicates that STZ-induced diabetic pathologies illicit a faster response to capsaicin, independent of the magnitude of response or percentage of capsaicin responsive cells.

## Discussion

In this study, we outline a simple and effective method to induce diabetic neuropathy pathologies in male and female mice. While STZ-induced DPN has historically been male biased, we report here that i.v. STZ induces diabetic pathologies in male and female mice as characterized by significantly increased fed blood glucose levels and significant glucose tolerance impairment. These results model the dysglycemia present in clinical populations diabetic patients^38^. Additionally, we show that i.v. STZ-treated mice of both sexes develop mechanical hypersensitivity, thereby modeling painful diabetic neuropathy pathology.

Furthermore, to our knowledge, we show for the first time that i.v. administration of STZ induces painful and nonpainful variants of diabetic neuropathy pathology in both male and female mice. The presence of these phenotypes models the clinical presentation of diabetic neuropathy^13^. While we found no significant difference in fed glucose levels between male mice with painful-DPN or nonpainful-DPN pathology, we do show that female mice displaying a nonpainful DPN-pathology had significantly lower glucose levels than mice with painful DPN-pathology. Additionally, we observed a positive correlation between fed blood glucose levels and mechanical hypersensitivity in females, indicating that hyperglycemia may be directly related to painful neuropathy in females but indirectly related in males.

The distinct hyperglycemic profiles we observed in females can be attributed to the presence of female sex hormones, as estrogen has a prominent role in glucose homeostasis and metabolism^39^. Estrogens have been shown to be protective against diabetic onset in female rodents in T1DM, and T2DM models^40,41^, and studies in clinical populations further indicate that female gonadal hormones have a protective role in the onset of diabetes^39,42^. The positive correlation between fed glucose levels and mechanical hypersensitivity can provide support for the postulation that hyperglycemia is a primary driver of nerve injury in type 1 DM^43,44^. Future studies targeting mechanisms that drive STZ-induced hyperglycemia in females should consider whether changes in estrous cycling or gonadal hormones accompany the distinct hyperglycemic hormones observed following i.v. STZ administration.

Several possible mechanisms may underly STZ-induced changes in evoked pain measures. Therefore, changes in transient calcium signaling may be helpful in further understanding how diabetic pathologies influence neuronal signaling. We found that DRG neurons from both STZ treated male and female mice had a lower latency to peak amplitude in response to capsaicin, indicating that TRPV1 receptors are implicated in impaired neuronal signaling. To our knowledge, this is a novel finding on the temporal resolution of transient calcium activity in response to capsaicin in a DPN murine model.

A limitation of this study is that we were unable to assess histological markers to further elucidate mechanisms underlying painful and nonpainful DPN. We aimed to identify a method that could easily induce diabetic pathology in male and female mice; however, assessment of histological markers in future studies would help further understand the mechanisms driving diabetic neuropathy.

## Conclusion

We believe that this study provides a simple and effective method to induce diabetic pathology in males and females using STZ. Our results provide novel insight on the relationship between hyperglycemia and neuropathic pain in males and females and underscores the importance of the consideration of sex as a biological variable. Additionally, our study further asserts the need for continued study on the mechanisms underlying the development of DPN.
